# A Case of Severe Proton Pump Inhibitor-Induced Hypomagnesemia Refractory to Continuous Oral and Intravenous Magnesium Replenishment

**DOI:** 10.7759/cureus.54483

**Published:** 2024-02-19

**Authors:** Karim Kheir, Omar Al Jassem, Georgio El Koubayati, Fady Haddad

**Affiliations:** 1 Department of General Medicine, Faculty of Medicine, Lebanese University, Beirut, LBN; 2 Department of Internal Medicine and Clinical Immunology, Faculty of Medicine, Lebanese University, Beirut, LBN; 3 Department of Internal Medicine and Clinical Immunology, Lebanese Hospital Geitaoui - University Medical Center, Beirut, LBN

**Keywords:** proton-pump inhibitors (ppis), hypomagnesemia, electrolyte imbalance, magnesium homeostasis, intestinal malabsorption

## Abstract

Proton pump inhibitors (PPIs) are frequently used medications to treat a wide variety of gastrointestinal conditions. By irreversibly inhibiting the hydrogen-potassium ATPase pump, they remarkably reduce gastric acid secretion. However, chronic PPI intake can result in serious complications, including severe hypomagnesemia. The following case report presents a severe case of refractory PPI-induced hypomagnesemia (PPIH), resistant to continuous oral and intravenous magnesium replacement, in a 70-year-old male patient, with a long history of PPI use due to persistent epigastric pain. Upon each of the 10 admissions to the hospital, he presented with severe signs and symptoms of hypomagnesemia, such as nausea, muscle fasciculation, diffuse cramps, weakness, neuromuscular irritability, and ECG disturbances, including non-specific T-wave abnormalities. In fact, PPIH has been reported for the first time in 2006. It is believed that the excessive, chronic intake of PPIs can disturb the normal functioning of the transient receptor potential melastatin 6/7 (TRPM 6/7), which is the main pathway of active intestinal magnesium absorption, leading to hypomagnesemia. PPIH is typically characterized by stubborn resistance to oral and intravenous magnesium replenishment but usually resolves after PPI withdrawal. Hence, despite being among the safest and most commonly prescribed drugs, PPI intake should be closely monitored when prolonged usage is planned. Additionally, continuous follow-up and regular assessment of serum magnesium levels are crucial to avoid the occurrence of PPIH and to prevent its potentially deleterious complications, including life-threatening arrhythmias.

## Introduction

Proton pump inhibitors (PPIs) constitute a class of drugs used to suppress the production of gastric acid. As of 2015, the United States Food and Drug Administration (FDA) recognizes six PPIs: omeprazole, esomeprazole, lansoprazole, dexlansoprazole, pantoprazole, and rabeprazole [1]. In fact, PPIs are considered to be among the most commonly prescribed drugs, and their use has revolutionized the management of acid-related disorders, including esophagitis, gastroesophageal reflux disease, peptic ulcer disease, Zollinger-Ellison syndrome, and Helicobacter pylori infections when combined with antibiotics [1].

PPIs achieve their therapeutic effect by binding to the luminal surface of the hydrogen-potassium ATPase pump (H+/K+ ATPase pump) on the parietal cells of the stomach [2]. Upon absorption in the gastric canaliculi, PPIs undergo activation and irreversibly bind to the H+/K+ ATPase pump, resulting in the permanent inactivation of this ATPase enzyme and disrupting the final step of gastric acid secretion [2].

The escalating global prevalence of PPI use underscores their therapeutic significance. However, this popularity raises concerns about potential risks associated with prolonged usage of PPIs, such as higher risks of respiratory and gastrointestinal infections; bone fractures, renal, hepatic, and cardiovascular diseases; and electrolyte imbalances, especially hypomagnesemia [3].

Several studies have implicated PPIs in disturbing magnesium homeostasis, leading to hypomagnesemia [3,4], but the underlying mechanisms and optimal management strategies remain unclear.

This case report aims to contribute to the existing literature by presenting a detailed analysis of a challenging clinical scenario, in which a patient presented with PPI-induced hypomagnesemia (PPIH), refractory to continuous oral and intravenous (IV) magnesium replenishment.

## Case presentation

We present hereby the case of a 70-year-old male patient with multiple comorbidities: hypertension, coronary artery disease, dyslipidemia, type II diabetes mellitus, Parkinson’s disease, and polyarteritis nodosa (PAN), with a long history of PPI use.

Due to a previous diagnosis of PAN, the patient was on chronic deflazacort therapy for his condition, after which he started experiencing severe steroid-induced epigastric pain. As a result, he was started on PPI therapy, which he took incessantly for a period of eight years, and alternated between the use of omeprazole, esomeprazole, and rabeprazole, based on availability.

The patient was followed up intermittently at the Lebanese Hospital Geitaoui - University Medical Center (LHG - UMC) for various reasons, during a period of 15 months, from December 2021, until February 2023, where he had 10 admissions to the hospital. The admission dates, as well as the reasons for hospitalization, are summarized in Table 1.

**Table 1:** The dates of admission and the reasons for hospitalization, during the last 10 admissions to the LHG - UMC.

Dates of admission (month/day/year)	Reasons of admission
12/13/2021	Fever and bronchopneumonia
12/27/2021	Fever and bronchopneumonia
03/05/2022	Nausea, vomiting, and dehydration
03/21/2022	Nausea and vomiting
04/04/2022	Gastric ulcers
06/11/2022	Gastroenteritis
09/28/2022	Anemia, chronic gastritis, and esophagitis
01/07/2023	Malaise and fatigue
01/12/2023	Urinary tract infection
01/25/2023	Pneumonia due to COVID-19

Upon each admission, the patient had, in addition to the symptoms that he presented with, severe signs and symptoms of hypomagnesemia, including nausea, muscle fasciculation, diffuse cramps, weakness, neuromuscular irritability, and ECG disturbances, including non-specific T-wave abnormalities. Consequently, electrolyte levels, particularly serum magnesium concentrations, were closely monitored during each admission. While hypomagnesemia is usually associated with hypermagnesiuria due to renal magnesium wasting, our patient presented with severely low urinary magnesium levels. No significant abnormal findings were detected in the serum or urine concentrations of other electrolytes.

**Figure 1:**
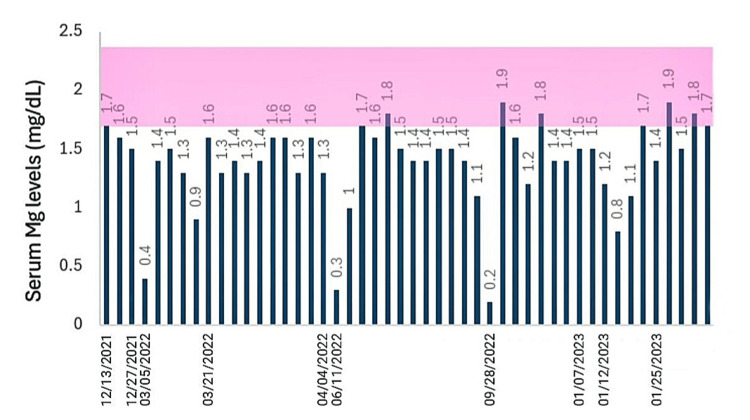
The patient's low serum Mg levels, measured during the last 10 hospitalizations.

The following figure (Figure 1) shows 48 readings of serum magnesium (Mg) levels during the last 10 admissions, knowing that the normal serum Mg levels, as shown in the highlighted pink area, range between 1.7 and 2.4 mg/dL [5].

According to the severity of the signs and symptoms of hypomagnesemia, and based on the serum Mg concentrations, several trials were made in order to correct the patient’s hypomagnesemia, using IV ampoules and oral tablets, as shown in Table 2.

**Table 2:** The range of serum Mg levels and the amount of Mg used to correct hypomagnesemia during each admission. *Normal range of serum Mg: 1.7-2.4 mg/dL **Each ampoule, given intravenously, contains 1 g of magnesium sulfate heptahydrate, or approximately 98.6 mg of elemental Mg. ***Each oral tablet contains 470 mg of magnesium lactate dihydrate or approximately 48 mg of elemental Mg.

Admission dates	Days of hospitalization	Mg levels during admission (mg/dL)*	Total Mg replenishment
12/13/2021	5	1.6-1.7	None
12/27/2021	3	1.5	None
03/05/2022	5	0.4-1.5	7 ampoules**
03/21/2022	13	1.3-1.6	11 ampoules + 6 tablets***
04/04/2022	1	1.3	None
06/11/2022	12	0.3-1.8	35 ampoules + 8 tablets
09/28/2022	11	0.2-1.9	19 ampoules + 6 tablets
01/07/2023	4	1.5	2 ampoules
01/12/2023	1	0.8-1.7	8 ampoules + 3 tablets
01/25/2023	8	1.4-1.9	10 ampoules

However, despite close follow-up and incessant oral and IV magnesium administration, the patient’s serum Mg levels were still below the normal range, or at the lower normal limits, emphasizing the severity of this rebellious case of PPIH.

## Discussion

Mechanism of action of PPIs

PPIs are widely used prodrugs, whose mechanism of action depends on both the physiology and the structure of the gastric H+/K+ ATPase pump [6]. Some ligands, such as acetylcholine, histamine, and gastrin, can bind to this ATPase enzyme, activate it, and cause it to release hydronium ions [7]. PPIs prevent this acid secretion by directly binding to and irreversibly inhibiting the H+/K+ ATPase pump [8].

Since PPIs are acid-activated prodrugs, a double protonation of PPIs in the secretory canaliculi of the activated parietal cells is essential to convert them into their active form [9]. This resultant, highly reactive thiophilic drug binds covalently to the α subunit of the H+/K+ ATPase pump to form a disulfide bond, preventing the hydronium ions from reaching the gastric lumen, through irreversible inhibition of the ATPase enzyme [10].

PPIs: benefits vs risks

PPIs are considered to be weak bases: this chemical property forces them to leave the blood and reach higher concentrations in the acidic medium of the secretory canaliculi, where the pH level is approximately one [10]. As a matter of fact, it has been shown that PPI concentration at the luminal surface of the H+/K+ ATPase pump is 1,000 times greater than their concentration in the blood, which majorly contributes to their therapeutic effect [10].

Hence, PPIs are generally considered to be safe and highly effective medications: they accumulate selectively at their target site of action, are only activated at low pH levels, are better suppressants of gastric acid secretion than histamine H2-receptor antagonists, and have an inhibitory activity that remarkably exceeds their plasma half-life [2].

However, this large window of safety does not come without adverse events: PPIs have been linked to serious electrolyte disturbances, particularly hypomagnesemia [11], as seen in the case of our patient.

As PPI-induced hypomagnesemia was first described in 2006, the underlying mechanisms of this relatively newly reported side effect have not been fully established yet; however, magnesium malabsorption due to disturbed activity of the transient receptor potential melastatin 6/7 (TRPM6/7) is believed to be at the origin of PPIH [12]. In fact, TRPM6 channels are the main pathway of active Mg2+ absorption in the intestines and are essentially stimulated at lower pH levels [12]. Nevertheless, prolonged PPI use can significantly alter the normal intestinal pH levels, which interferes with the normal activity of the TRPM6 channels. Consequently, a reduced affinity of the TRPM6 proteins to Mg2+ ions due to chronic PPI intake is directly linked to hypomagnesemia. This phenomenon can be exacerbated by genetic predispositions in individuals with particular common single nucleotide polymorphisms in the TRPM6 gene, leading to a 5.8-fold increase in the risk of developing hypomagnesemia, among patients with chronic PPI use [13].

Moreover, the reduction in Mg2+ solubility due to higher pH levels, and the subsequent decrease in Mg2+ absorption, are believed to play an important role in the pathophysiology of PPIH [11]. In particular, every two-unit increase in the pH of the gastric milieu, due to prolonged PPI intake, is directly associated with a one-unit increase in the pH of the small intestine [14]. At higher pH levels, Mg2+ ions have a greater affinity for binding negatively charged ions, such as Cl- and PO43-, causing a great decrease in the Mg2+ solubility and availability for intestinal absorption [11].

Furthermore, while certain drugs, such as cisplatin and calcineurin inhibitors, can induce hypomagnesemia due to increased renal wasting, this is not the case in PPIH, where affected patients typically present with very low urinary Mg2+ levels, indicating a renal compensatory mechanism [15]. These findings support the fact that PPIH is strictly due to an intestinal involvement [15].

PPIH: a rebellious case resistant to Mg replacement

In the case of our patient, after reviewing the multiple comorbidities and the drug history, it was first thought that the hypomagnesemia could be possibly explained by either an acute renal dysfunction or the hydrochlorothiazide intake, both of which can provoke low serum magnesium levels [16,17]. Nevertheless, hypomagnesemia in such cases is associated with renal magnesium wasting, while our patient had markedly low urinary Mg levels: after a 24-hour urine collection was done, lab tests showed a severe hypomagnesuria of 6.4 mg/24 hour, knowing that the normal urinary Mg concentrations, according to the reference values of our lab, range between 51 and 269 mg/24 hour.

Moreover, according to PubMed literature, no cases of hypomagnesemia were reported with any of the other medications taken by our patient, such as losartan, rivaroxaban, atorvastatin, gliclazide, sitagliptine, and carbidopa-levodopa. In view of the foregoing reasons, a high suspicion of PPIH was raised. This proposition was later confirmed by multiple episodes of rapid normalization of serum Mg levels upon PPI withdrawal, and the brisk recurrence of hypomagnesemia following several attempts at PPI resumption.

In addition, the incessant oral and IV Mg replenishment only minimally improved our patient’s serum Mg levels, when PPIs were still used. For instance, during the sixth admission, our patient was given the greatest doses of oral and IV Mg compared to any other admission (35 IV ampoules and eight oral tablets of magnesium during a period of 12 days, as shown in Table 2). Nevertheless, satisfactory serum Mg levels could not be reached, and the highest Mg concentration ever recorded during this admission was 1.8 mg/dL, a value considered to be at the lower limit of the normal range.

Moreover, on 6/18/2023, the patient’s serum Mg level remained unchanged (1.4 mg/dL), despite being given 6 g of IV MgSO4 the day before; however, a lesser dose of only 4 g of IV MgSO4 per day typically ensures the maintenance of serum Mg levels above 2 mg/dL [18].

After all these attempts to correct this PPIH showed unsatisfactory outcomes, complete PPI withdrawal was adopted, and total normalization of serum Mg levels was reached, as the patient’s lab tests showed a Mg level of 2.4 mg/dL following the last discharge. These findings confirm that the gold standard to control PPIH remains the complete withdrawal of PPIs whenever possible, or the use of PPIs in smaller doses and lesser frequency.

Indeed, we believe that the long history of PPI intake, coupled with the high dosage (40 mg/day, even 40 mg twice a day on certain occasions), was the main culprit accountable for this case of PPIH. Continuous follow-up and repeated measurements of the serum Mg levels were maintained throughout the 10 episodes of hospitalization and were encouraged, along with a complete cessation of PPI intake, upon each discharge from the hospital.

## Conclusions

Over the last decade, an increased interest in PPI-induced hypomagnesemia has developed. Since PPIH can result in deleterious clinical complications, including life-threatening arrhythmias, a regular follow-up, along with correction of serum Mg levels, is crucial. Obtaining a baseline Mg level in patients in whom prolonged PPI intake is planned remains of significant benefit for further follow-up. A reduction in the frequency and the dose of PPIs is highly encouraged, and the consideration of switching to histamine H2-receptor antagonists or antacids, in case of PPIH, should be taken whenever possible.
